# Supplementary figures and images for: Similarity-based metric analysis approach for predicting osteogenic differentiation correlation coefficients and discovering the novel osteogenic-related gene FOXA1 in BMSCs
Source: PeerJ. 2024 Sep 19;12:e18068. doi: 10.7717/peerj.18068 (PMC11416762; doi:10.7717/peerj.18068)

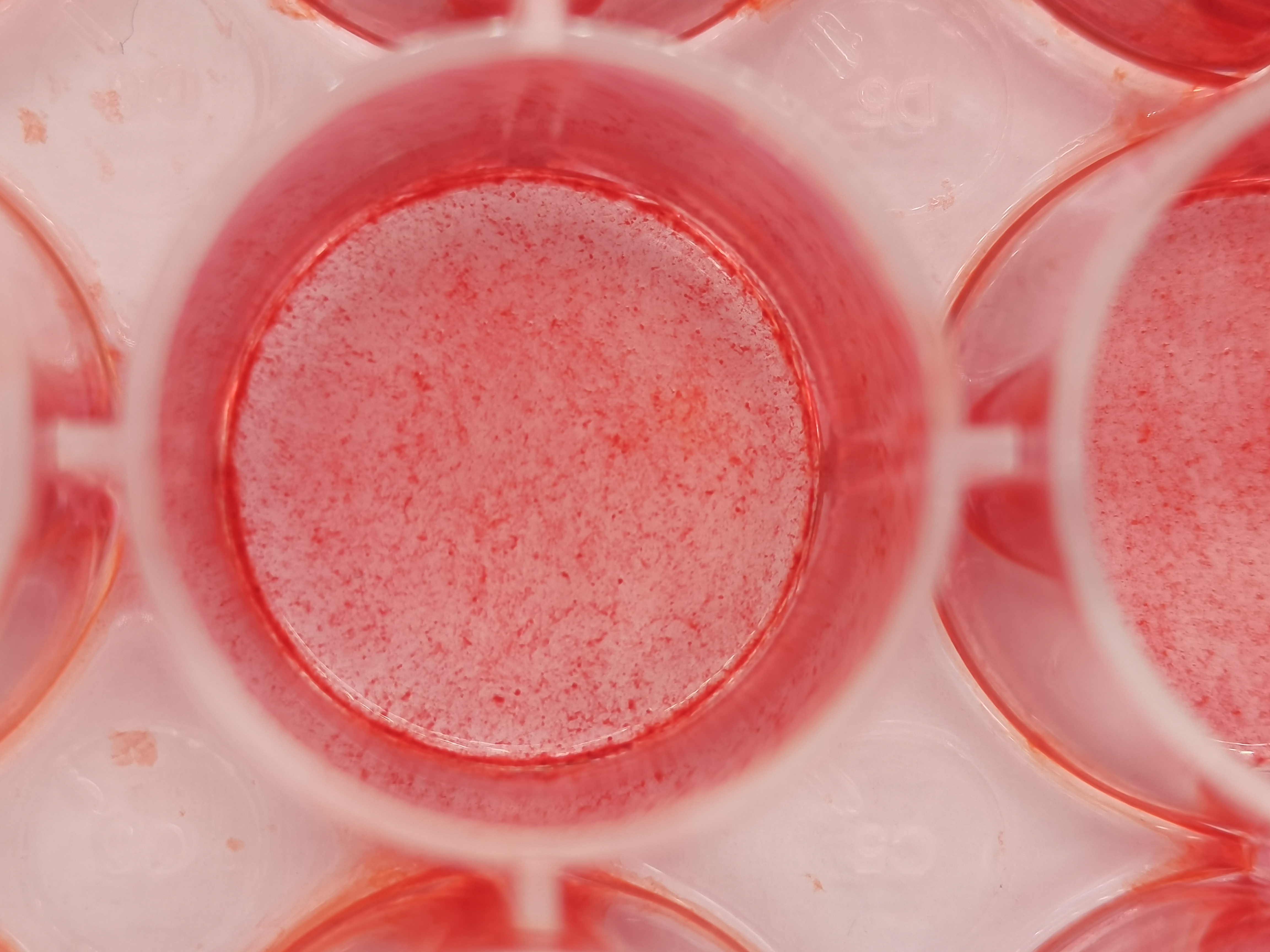

Supplement: Supplemental Information 1 [file peerj-12-18068-s001.zip › 1.jpg]

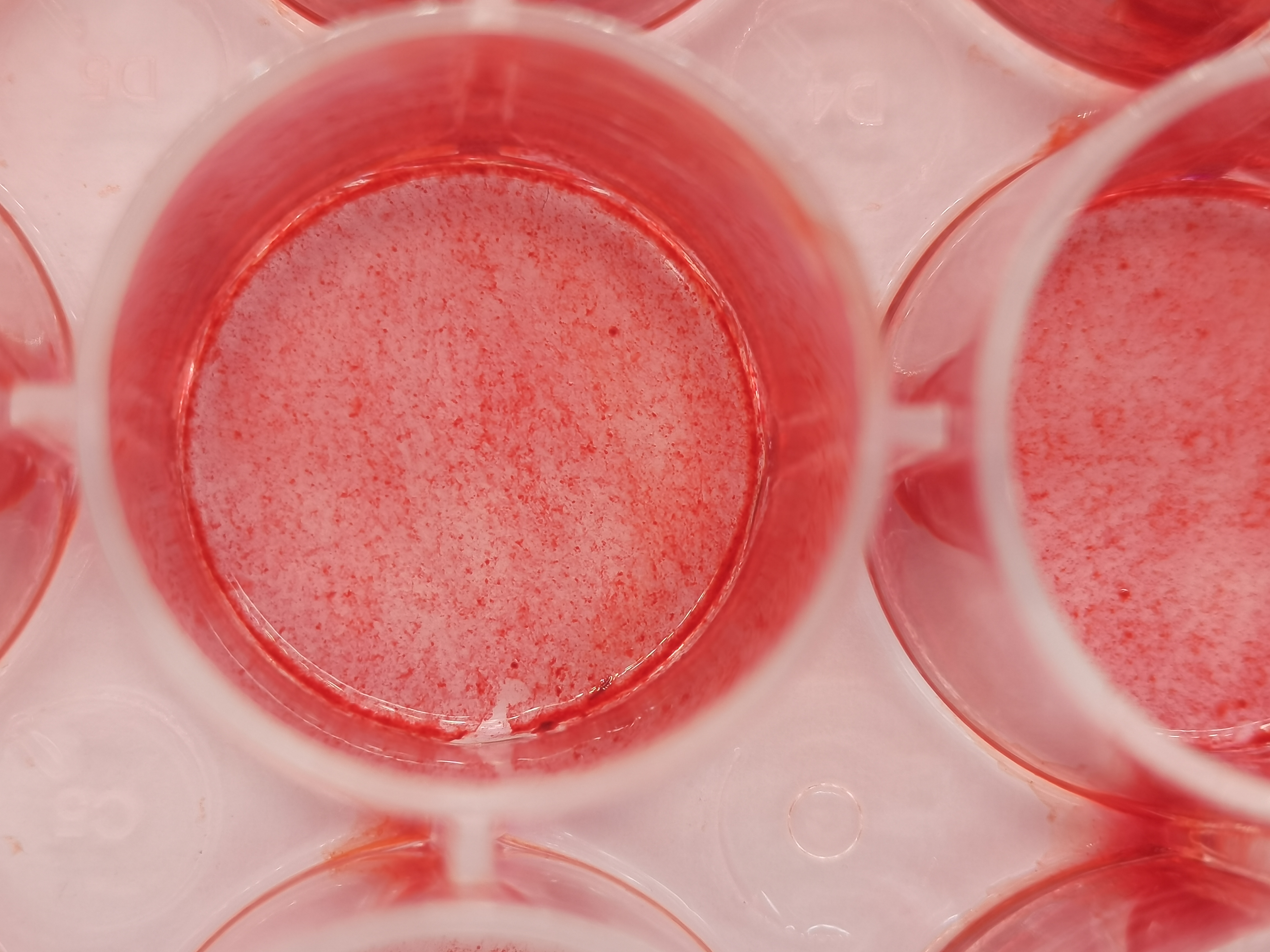

Supplement: Supplemental Information 1 [file peerj-12-18068-s001.zip › 2.jpg]

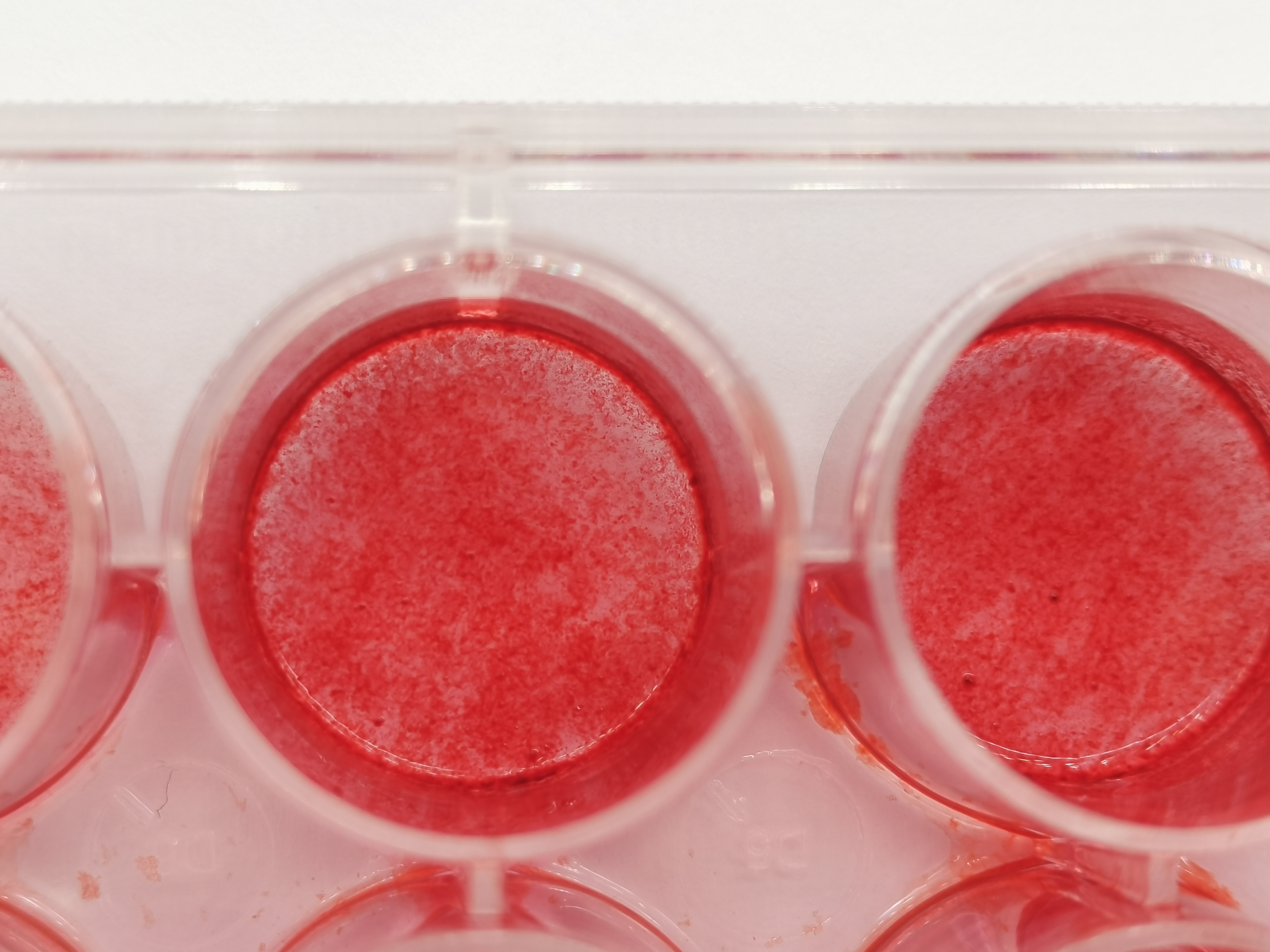

Supplement: Supplemental Information 1 [file peerj-12-18068-s001.zip › 3.jpg]

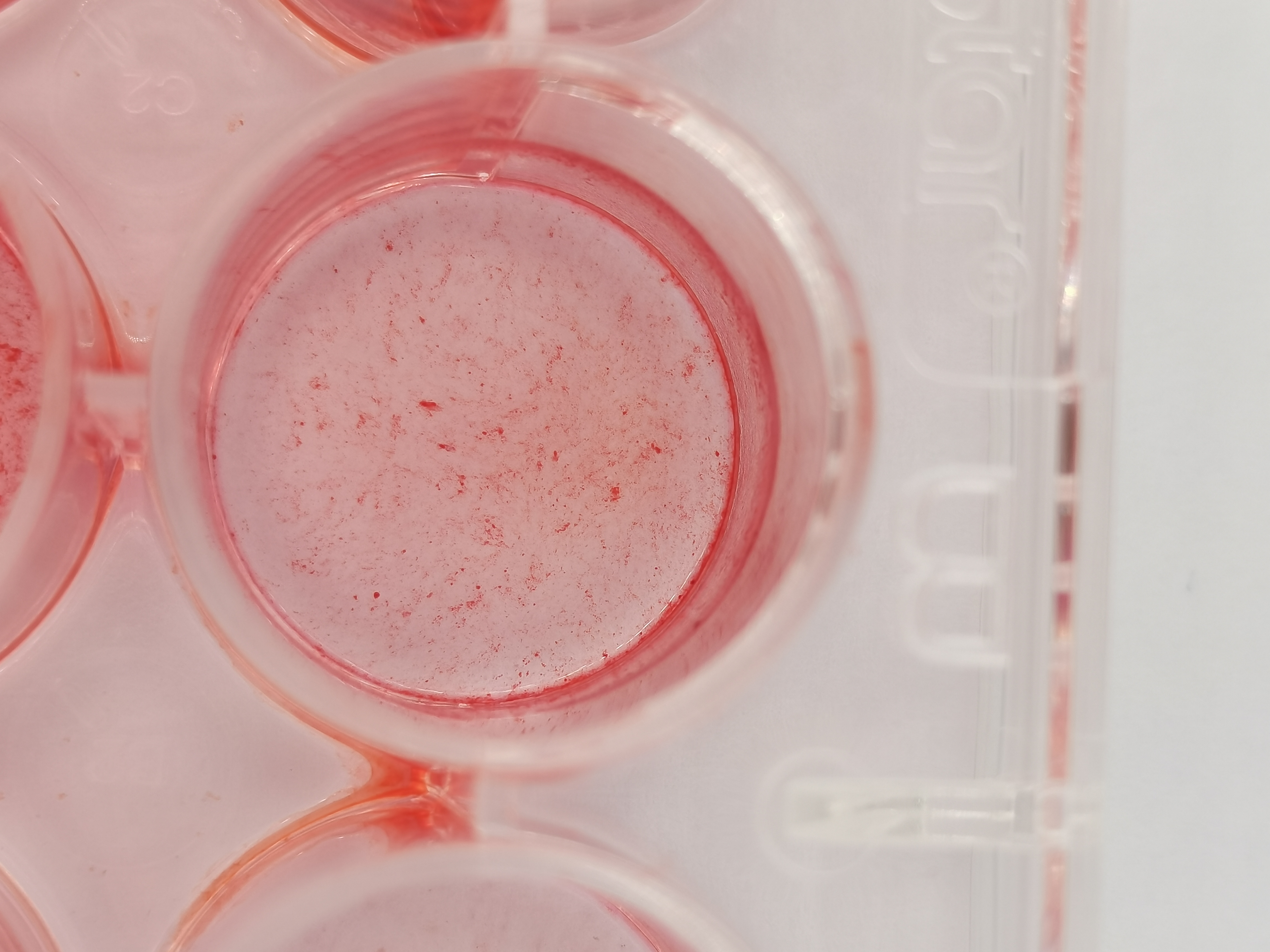

Supplement: Supplemental Information 1 [file peerj-12-18068-s001.zip › 4.jpg]

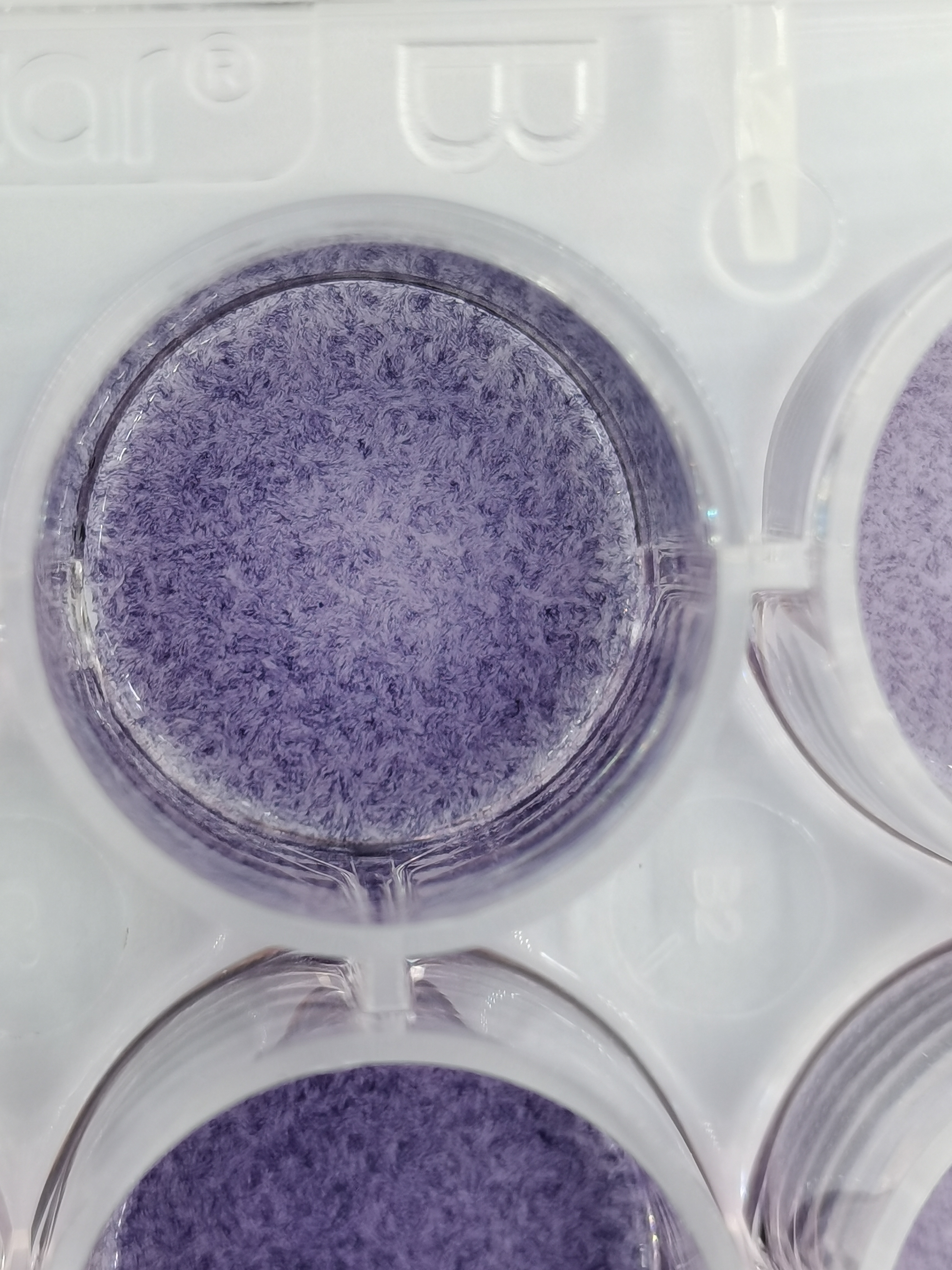

Supplement: Supplemental Information 1 [file peerj-12-18068-s001.zip › 5.jpg]

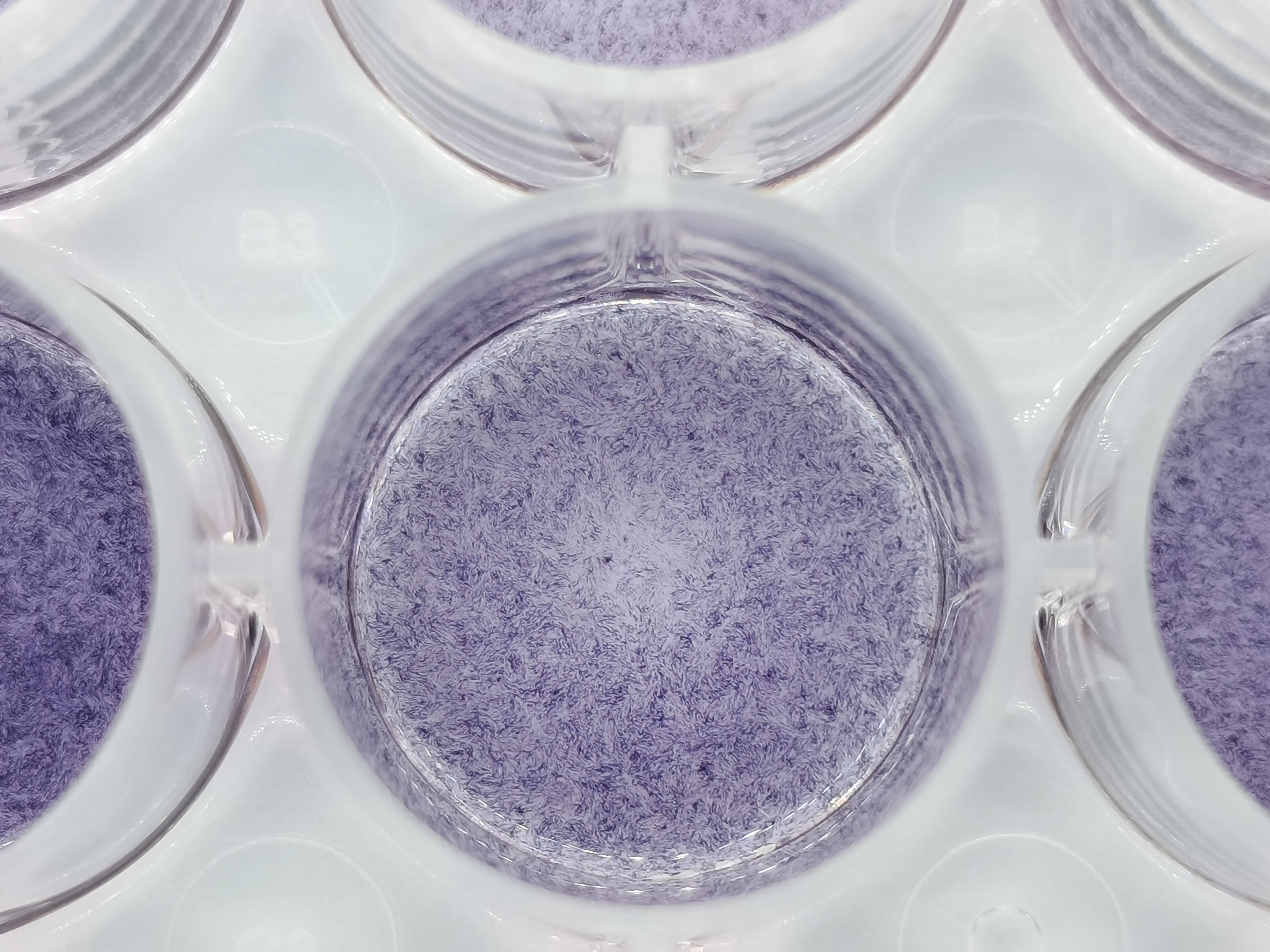

Supplement: Supplemental Information 1 [file peerj-12-18068-s001.zip › 6.jpg]

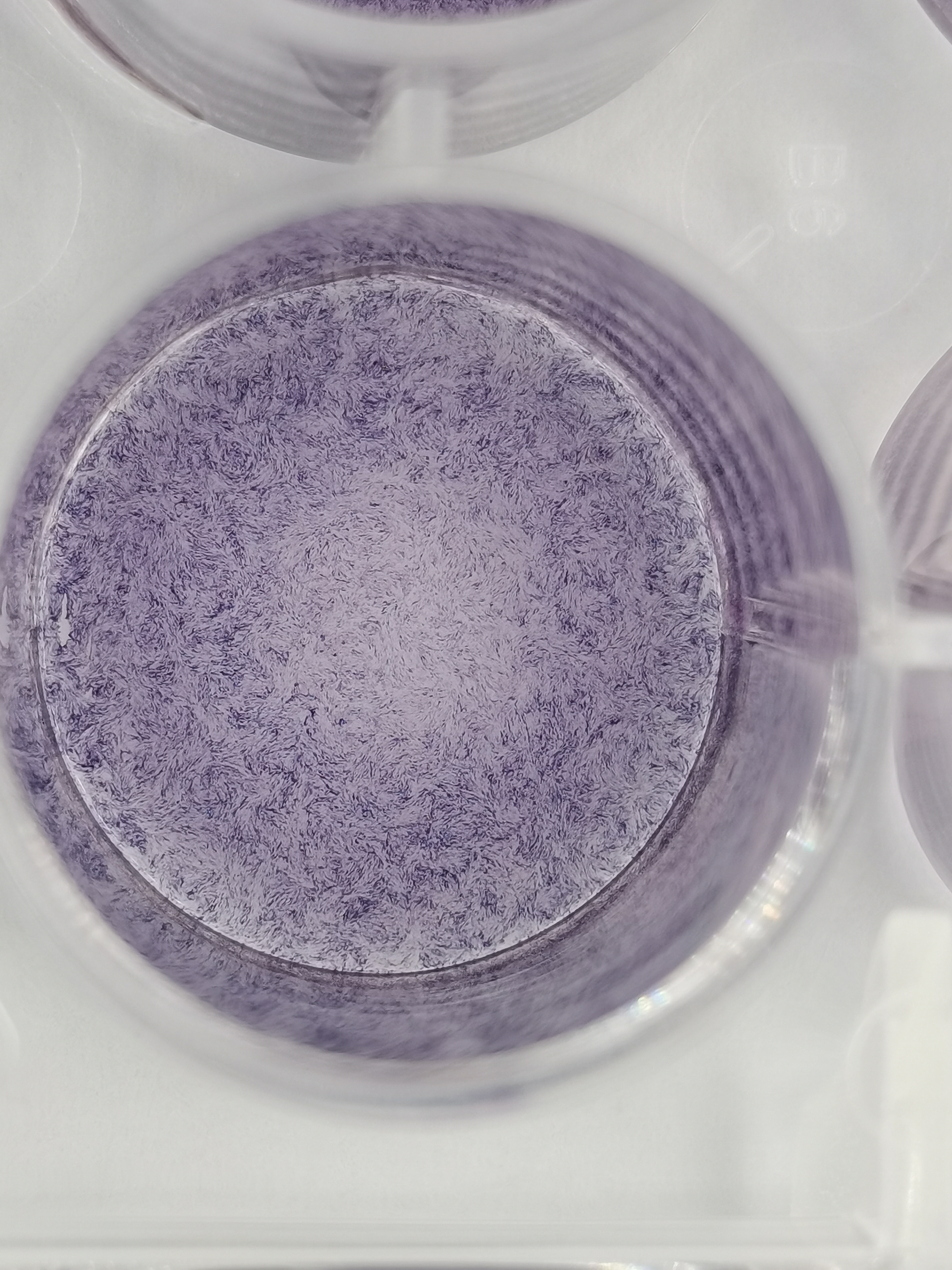

Supplement: Supplemental Information 1 [file peerj-12-18068-s001.zip › 7.jpg]

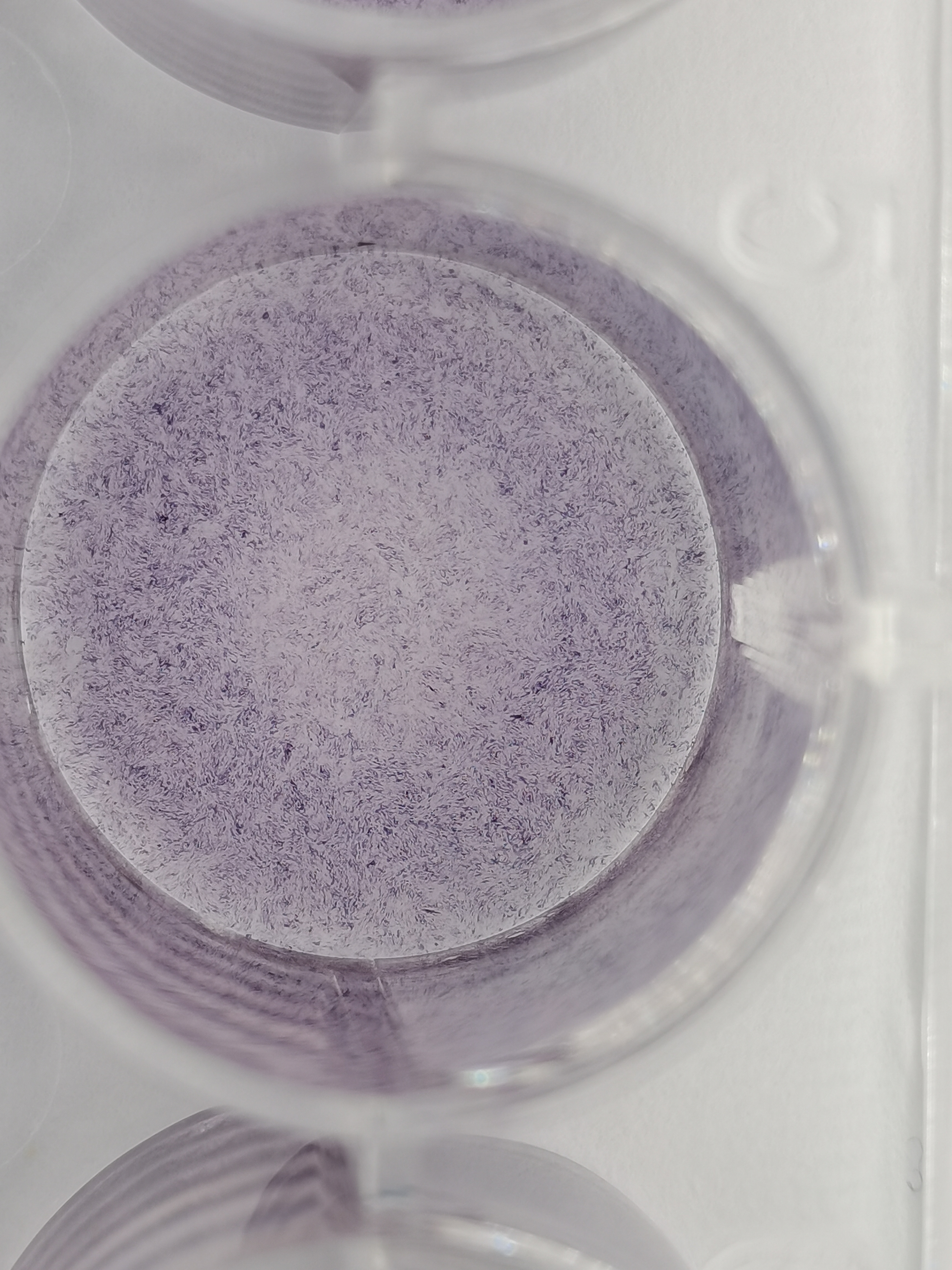

Supplement: Supplemental Information 1 [file peerj-12-18068-s001.zip › 8.jpg]

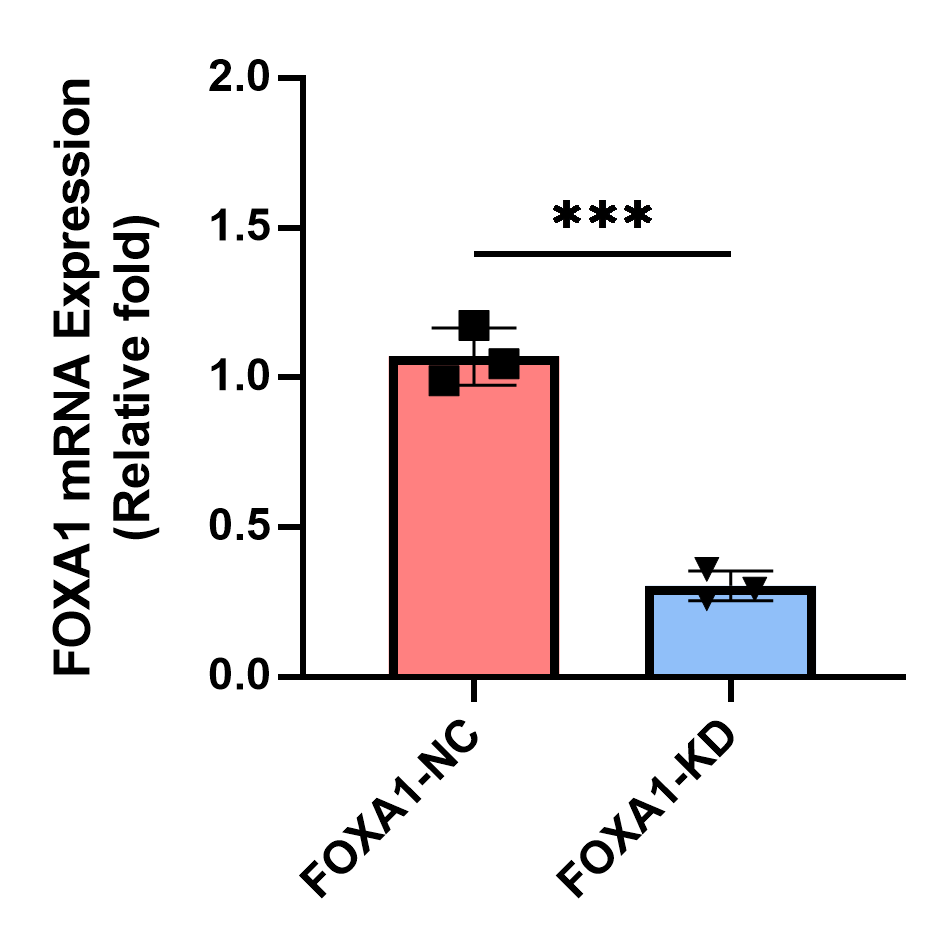

Supplement: Supplemental Information 4 [file peerj-12-18068-s004.tif]
