# Supplementary material for: Similarity-based metric analysis approach for predicting osteogenic differentiation correlation coefficients and discovering the novel osteogenic-related gene FOXA1 in BMSCs
Source: PeerJ. 2024 Sep 19;12:e18068. doi: 10.7717/peerj.18068 (PMC11416762; doi:10.7717/peerj.18068)
Supplement: Supplemental Information 3 — Human bone mesenchymal stem cells, product number: HUXMA-01001, Cyagen Biosciences, Guangzhou, China. [file peerj-12-18068-s003.pdf]

Machine Translated by Google

OriCell®

Product test report

OriCell® Adult Bone Marrow Mesenchymal Stem Cells

Product number: HUXMA-01001

We help you discover life

Machine Translated by Google

Basic Information

product name	OriCell® Adult Bone Marrow Mesenchymal Stem Cells
Part Number	HUXMA-01001
batch number	210406H61
Donor information	male
Cryopreservation passage	P2
Storage Conditions	Liquid nitrogen (-196°C)

Testing standards and results

Test items			Test results	Testing standards	in conclusion
often regulation Inspection Measurement	Bacteria, fungi		Negative	Negative	Passed
	Mycoplasma		Negative	Negative	Passed
	Endotoxins		ÿ10EU	ÿ10EU	Passed
Recommendation Certainly Inspection Measurement	Resuscitation survival rate		98.95%	ÿ80%	Passed
	Number of living cells		1.88×106	ÿ1×106	Passed
	Cell recovery and adhesion rate		99.52%	ÿ80%	Passed
	Growth status		The morphology is long and spindle-like, with polar arrangement.<br>The population doubling time was 17.96 h	The morphology is long fusiform and arranged in polar pattern;<br>Population doubling time ÿ 72 h	Passed
	surface noodle Standard remember point Child	CD105	90.04%	ÿ70%	Passed
		CD29	99.08%	ÿ70%	Passed
		CD73	99.11%	ÿ70%	Passed
		CD34	0.80%	ÿ5%	Passed
		CD45	0.91%	ÿ5%	Passed
		CD11b	0.80%	ÿ5%	Passed
		CD44	99.45%	ÿ70%	Passed

Machine Translated by Google

Cell growth status

Cell proliferation ability

The data of the logarithmic phase (1-2 days) were selected for calculation, and the cell population doubling time was found to be 17.96h.

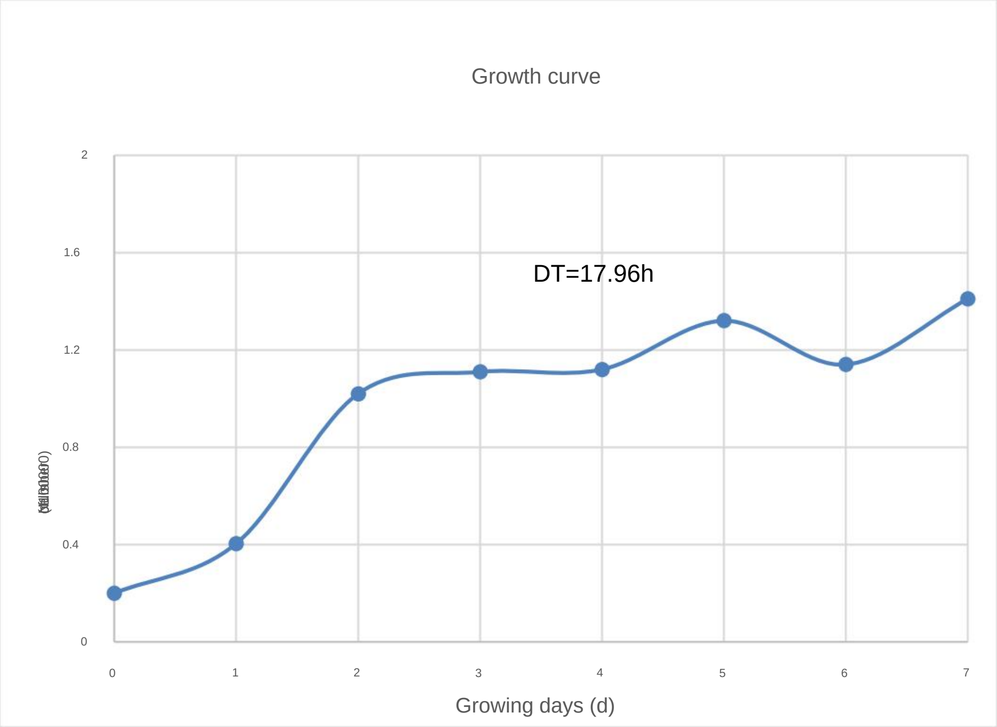

Machine Translated by Google

Cell passaging capacity

The cells all grew in a long spindle shape with good polarity and three-dimensional sense. After 5 passages, the cells were still quite active.

The morphology of this batch of cells after 72 hours of growth in the Pn+1 generation

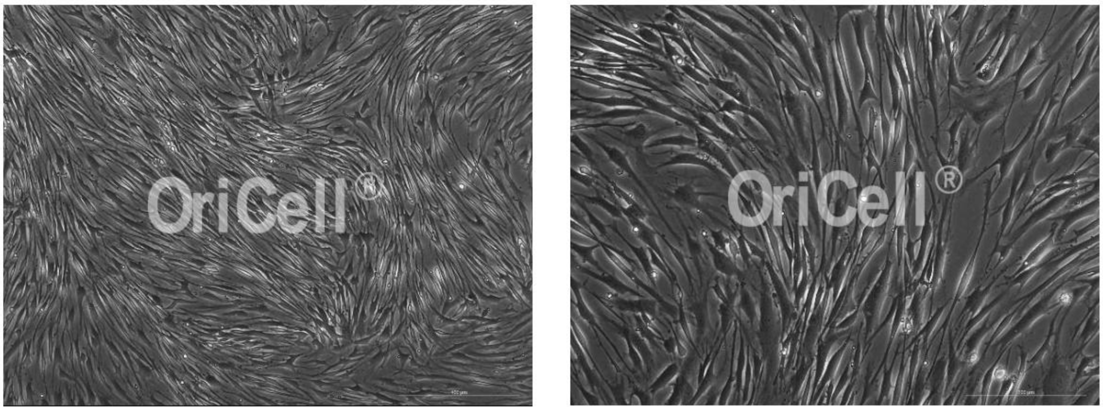

The morphology of this batch of cells after 48 hours of growth in the Pn+3 generation

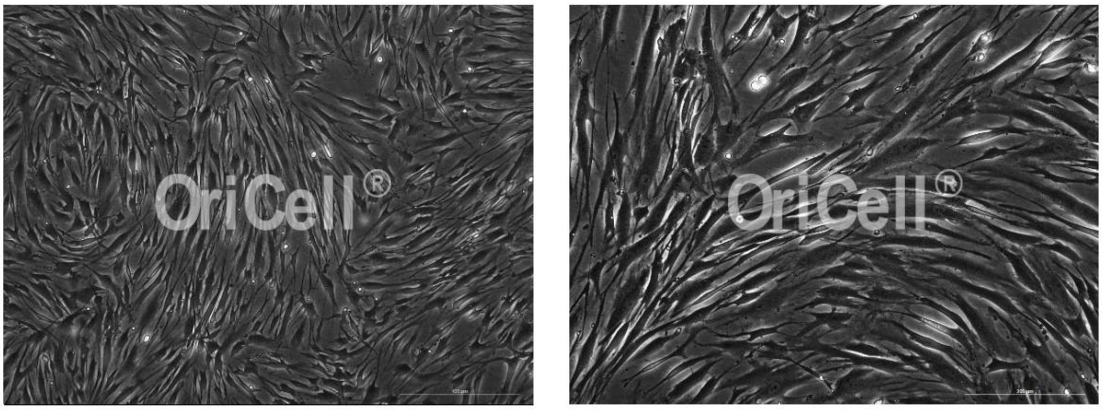

The morphology of this batch of cells after 48 hours of growth at generation Pn+5

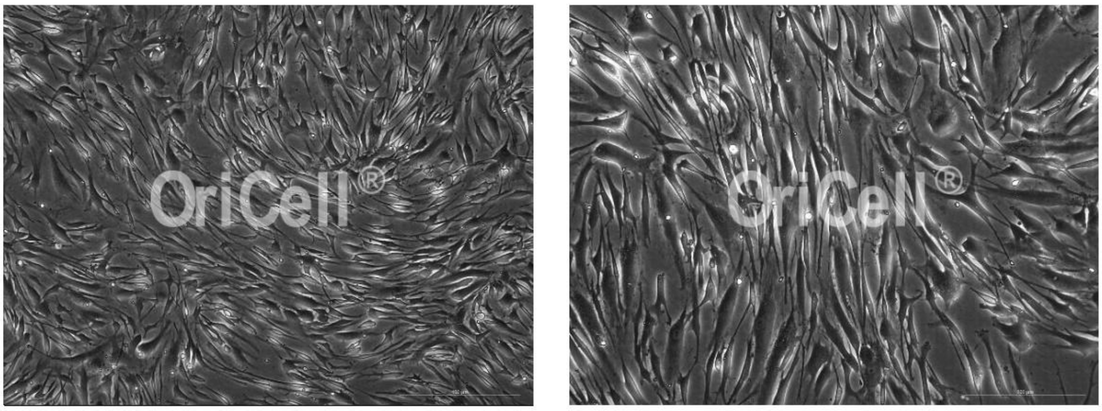

OriCell®

Induced differentiation ability

Adipogenic differentiation

When the cell confluence reached about 90%, mesenchymal stem cell adipogenesis induction medium was added. After 39 days, Oil Red O staining was performed, and red cells were visible.

More standard lipid droplets.

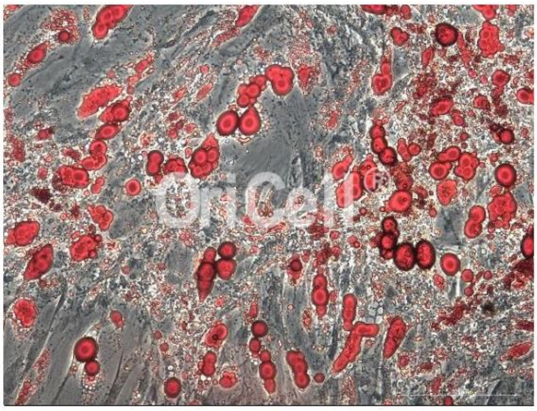
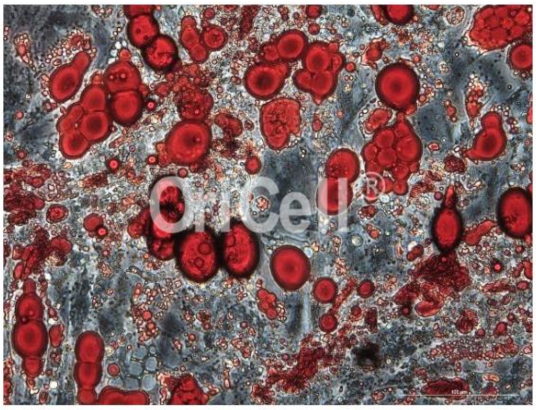

Osteogenic differentiation

When the cell confluence reached about 70%, the mesenchymal stem cell osteogenic induction solution was added, and Alizarin red staining was performed 35 days later.

Combined to form concentric circles of small dark red nodules.

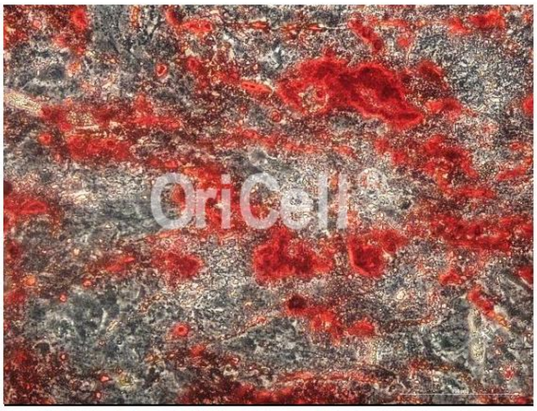
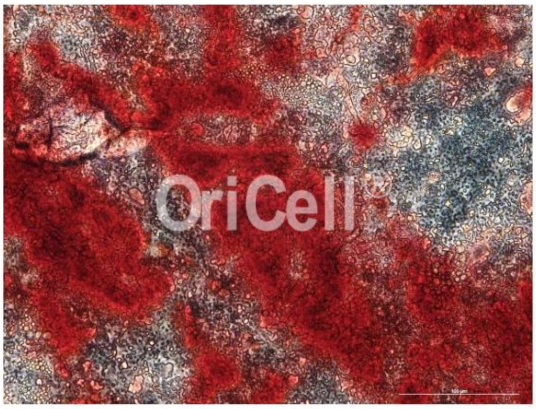

Machine Translated by Google

Chondrogenic differentiation

After cell mass culture and mesenchymal stem cell chondrogenic induction medium induction culture, the cells gradually changed from flat cell masses attached to the bottom of the centrifuge tube to cells.

After 21 days of induction, the cell spheres became larger and rounder, and the surface became smooth.

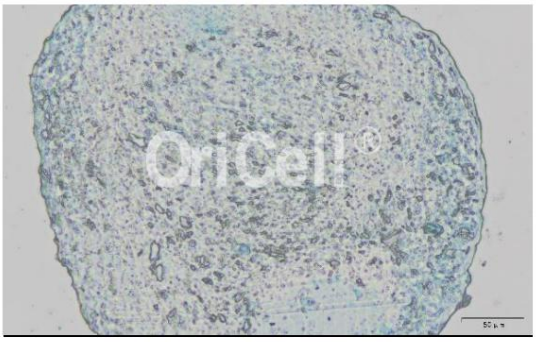

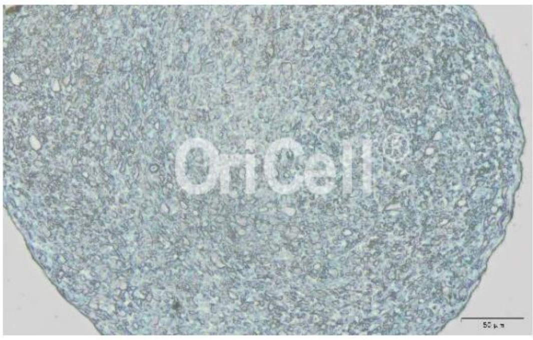

Machine Translated by Google

Surface molecular flow detection

Data Details

Mouse IgG1, ÿ Isotype Control Antibody

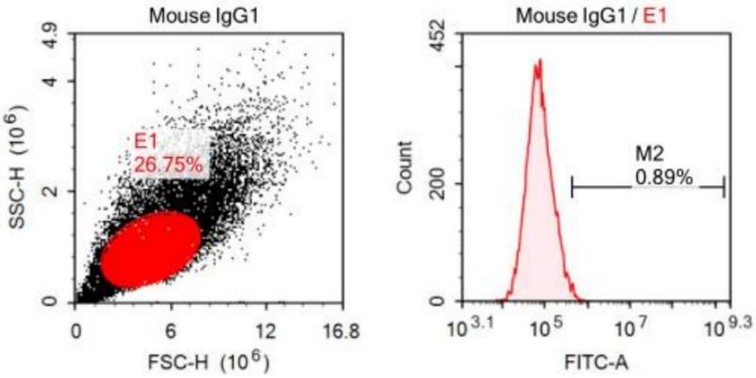

Anti-human CD105 antibody

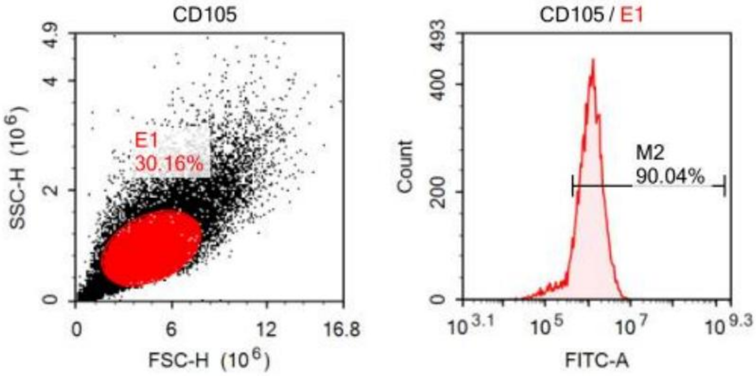

Anti-human CD29 antibody

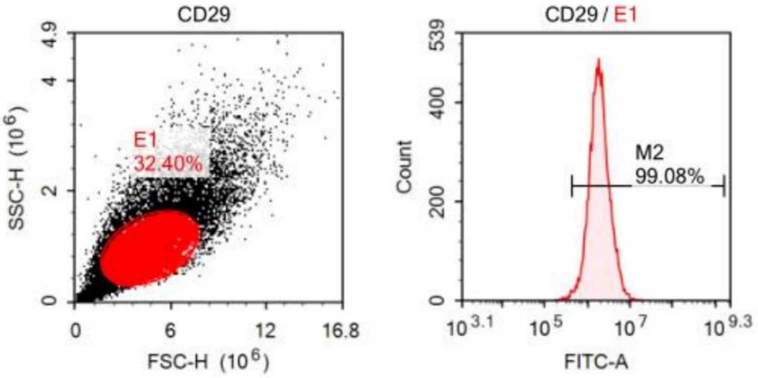

Anti-human CD73 antibody

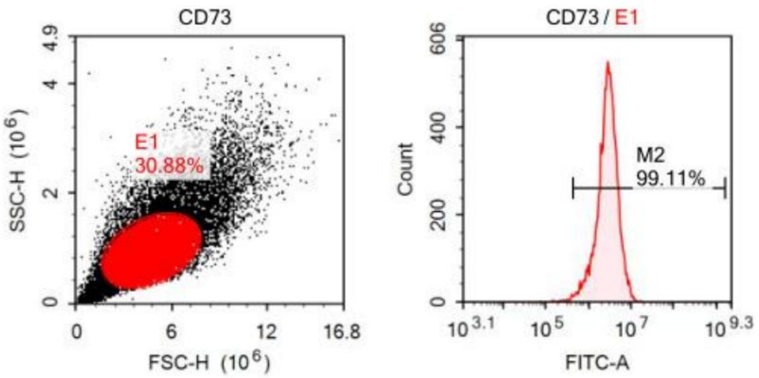

Anti-human CD34 antibody

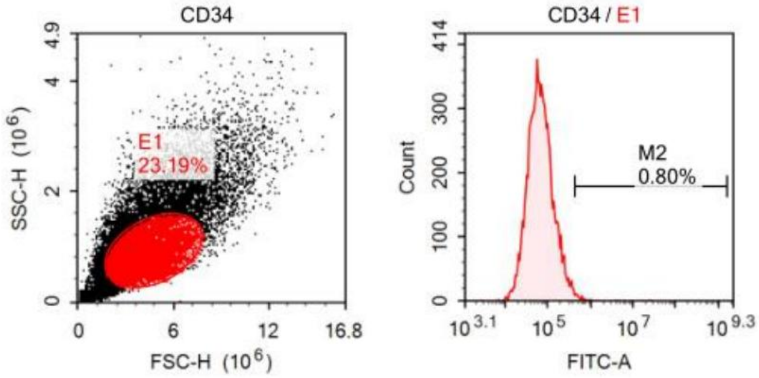

Anti-human CD45 antibody

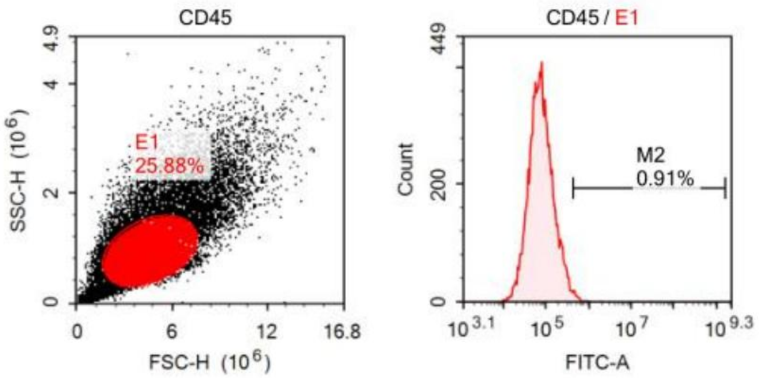

Anti-human CD11b antibody

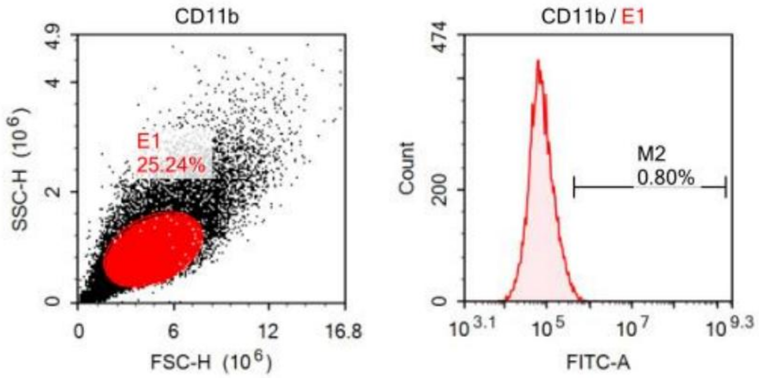

Machine Translated by Google

Rat IgG2b, ÿ Isotype Control Antibody

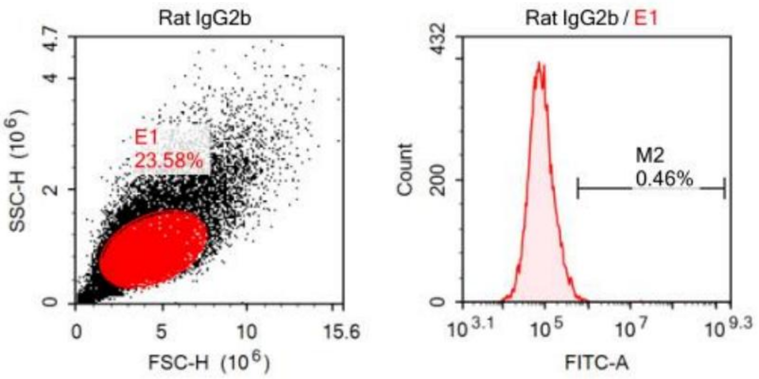

Anti-human CD44 antibody

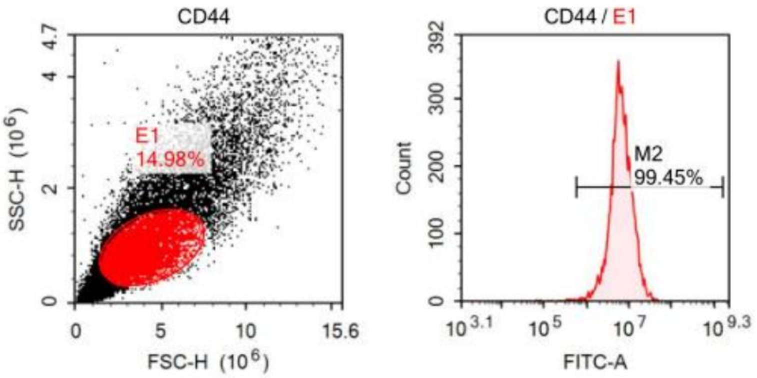

Saiye (Guangzhou) Biotechnology Co., Ltd. reserves all rights to the technical documents of OriCell® cell culture products.

Without the written permission of Saiye (Guangzhou) Biotechnology Co., Ltd., any part of this document may

It may not be adapted or reproduced for other commercial purposes.
